# Exploring Formal Caregiver Burden Within Nursing Homes: An Integrative Review

**DOI:** 10.1093/geroni/igaa057.489

**Published:** 2020-12-16

**Authors:** Rachel Kunkle, Claudia Chaperon, Ann Berger

**Affiliations:** University of Nebraska Medical Center, Omaha, Nebraska, United States

## Abstract

The purpose of this integrative review was to explore formal caregiver burden in nursing homes among direct care nursing staff. The aim was to gain an understanding of the state of the science of formal caregiver burden, it’s measurement, and its effect on resident care. Based on PRISMA guidelines, a systematic search of CINAHL, PubMed, PsycINFO and Embase databases was conducted using terms from the definition of formal caregiver burden, three conceptual models, and the thesaurus feature of each database for years ranging from 1979-2019. Inclusion criteria consisted of peer-reviewed articles in English that focused on the key terms of formal caregiver burden among direct care nursing staff only in nursing home facilities. Out of 925 citations, 20 articles met criteria; 15 quantitative and 5 qualitative studies. Sample sizes ranged from 11-1283, number of facilities from 1-55, and bed size from 31-203. Psychometric measures used in the studies reported lower validity and reliability, a variety of conceptual definitions interchangeably, and primarily studied nursing assistants (n=19). Five studies included nurses as formal caregivers, and one studied only nurses. Variables used to identify burden were racial disparities (n=2), trauma experience (n=1), depression (n=4), distress (n=3), mental health (n=1), stress (n=2), health complaints (n=1), and alienation (n=1). Two studies evaluated the effect on resident care, one finding significant results of in-service training contributing to positive attitudes of formal caregivers toward confused residents. Current limitations in the understanding of formal caregiver burden limit the advancement of research.

